# The growth rate of “clinically significant” renal cancer

**DOI:** 10.1186/s40064-015-1385-9

**Published:** 2015-10-06

**Authors:** Ofer N. Gofrit, Vladimir Yutkin, Kevin C. Zorn, Mordechai Duvdevani, Ezekiel H. Landau, Guy Hidas, Dov Pode

**Affiliations:** Department of Urology, Hadassah Hebrew University Hospital, POBox 12000, 91120 Jerusalem, Israel; Section of Urology, Department of Surgery, University of Montreal Health Center (CHUM), 235 Rene Levesque Est, Montreal, QC H2X 1N8 Canada

**Keywords:** Renal carcinoma, Growth rate, Cross-sectional imaging

## Abstract

Surveillance studies of enhancing renal masses report on a mean tumor growth rate of about 0.3 cm/year. In most of these studies however, only small tumors in elderly patients were followed. In the current report, we attempt to evaluate the growth rate of “clinically significant” renal carcinomas defined as tumors that were treated immediately upon diagnosis. 46 patients (mean age 64 years SD 11 years) were treated for renal carcinoma. All had a cross-sectional imaging studies performed 6–60 months prior to diagnosis of kidney cancer demonstrating no tumor. Tumor growth rate was calculated by dividing tumor’s largest diameter by the time interval between the normal kidney imaging and diagnosis of renal cancer. Mean tumor diameter was 4.5 cm (SD 2.4 cm). Mean time period from the normal imaging to diagnosis of renal cancer was 33.6 months (SD 18 months). According to the proposed model, the average growth rate of “clinically significant” renal carcinomas was 2.13 cm/year (SD 1.45, range 0.2–6.5 cm/year). Tumor growth rate correlated inversely with patient’s age (p = 0.007). Patient gender or Fuhrman’s grade did not correlate however. The growth rate of “clinically significant” renal cancer appears to be higher than the rate reported in surveillance trials. Renal tumors tend to grow faster in young patients. As such, variable growth rate should be taken into account when considering active surveillance in young patients and when designing trials for evaluation of anti-cancer agents.

## Background

The growth rate of solid tumors is an important parameter in understanding their biology, in designing neoadjuvant trials or when appreciating novel anti-cancer agents. The growth rate of “clinically significant” renal cancer is not well understood since patients with “significant tumors” are often treated without delays (Cambell et al. 2007). Most of the literature on growth rates of renal tumors is driven from surveillance studies in patients with solid enhancing renal masses. These are usually small tumors in elderly patients and in many cases without histologic confirmation. Collectively, slow growth rate of 0.3 cm per year in the largest diameter is reported in most of these studies. A meta-analysis of 10 manuscripts reporting on 234 patients with enhancing renal masses that were not treated upon diagnosis also demonstrated a small growth rate (Chawla et al. 2006). In that study, mean patient age was 71 years (the Fox Chace Cancer Center experience only), mean lesion size at presentation was 2.6 cm and mean follow-up period was 30 months. The growth rate of renal masses in that study was 0.28 cm/year (range 0.09–0.86 cm/year). A somewhat faster growth rate was observed in the subgroup of patients with pathologically confirmed renal cancer (0.4 cm/year, range 0.42–1.6 cm/year). In another large study by Jewett et al., a growth rate of 0.13 cm/year was reported (Jewett et al. 2011), and even when larger tumors are being followed growth rate did not exceed 0.6 cm/year (Mues et al. 2010; Mehrazin et al. 2014).

Unfortunately, generalizing the growth rate of renal tumors from studies of elderly, frail patients under active surveillance may be misleading. More specifically, these patients represent a selected population in which both the patient and the physician deem no significant survival impact by the renal tumor. Very few attempts have been made to assess the growth rate of “clinically significant” malignant renal tumors. In a single study of 9 patients with renal imaging 6 months or more prior to the diagnosis of renal cancer demonstrating no tumor or a small tumor that was overlooked, Staehler et al calculated an extremely high growth rate of 6.4 cm per year (Staehler et al. 2010). To better investigate this matter, we sought to evaluate a larger population of patients who were treated for renal cell carcinoma.

In this study we calculated growth rate of “clinically significant” renal tumors (that were treated shortly after diagnosis) using prior cross-sectional imaging study showing no renal mass.

## Methods

### Patients

A total of 435 patients underwent surgical treatment for renal cancer in our institution between January 1998 and December 2013. In 46 patients (mean age 64 years, SD 11 years) previous cross-sectional imaging studies of the urinary system were done 6–60 months prior to the diagnosis of the renal cancer showing no evidence of kidney cancer. Previous imaging studies included abdominal computerized tomography in 18 cases (10 with and 8 without intravenous contrast injection) and renal ultrasonography in 28 cases. Institutional review board approval was obtained (#202-5.9.08).

Mean time interval between the imaging studies showing no renal tumor and diagnosis of renal cancer was 33.6 months (SD 18.2 months). In 13 cases (28.2 %), the imaging study showed another renal pathology (renal cysts in 9 cases, stones in 3 and atrophic kidney in 2 cases). In the rest of the patients, normal kidneys were demonstrated. All patients were operated shortly after diagnosis. Partial nephrectomy was performed on 24 patients, radical nephrectomy upon 20 and radiofrequency percutaneous needle ablation in 2 patients. Median Post-operative follow-up was 68 months.

The pathological specimens were evaluated according to the 2002 version of the TNM classification (Greene et al. 2002), the histologic subtyping according to the 1997 UICC classification (Störkel et al. 1997), and the grading according to Fuhrman’s nuclear grading system (Fuhrman et al. 1982). Grading of clear cell cancers was dichotomized to low (Fuhrman’s grades 1–2) and high (Fuhrman’s grades 3–4) grades.

### Calculation of tumor growth rate

Calculation of tumor growth rate was based on two assumptions:

1. Macroscopic tumor growth commenced shortly after the normal imaging study.
2. Tumor growth was linear.

Annual tumor growth rate was calculated by dividing tumor’s largest diameter measured on the diagnostic computerized tomography by the time interval between the normal imaging studies to diagnosis of kidney tumor. The dependency of the growth rate on the following parameters was studied: patient’s age and gender, type of previous imaging study showing normal kidney (ultrasonography or computerized tomography), Fuhrman’s grade (Grades 1–2 vs. grades 3–4) and recurrence (patient’s that developed tumor recurrence vs. patients that did not). A 2-tailed the Student’s *t* test and analysis of variance were used for comparing the variables and a p value <0.05 was considered statistically significant. The JMP software (SAS Institute Inc. Cary, NC, USA) was used for data processing.

## Results

**Fig. 1:**
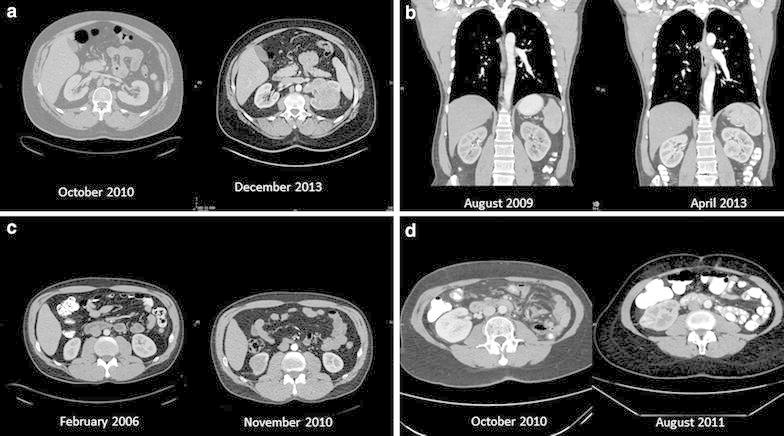
Computerized tomographic scans of selected patients. **a** A 52-year-old man, on October 2010 normal kidneys were found and on December 2013, a 9 cm clear cell tumor with IVC invasion was found (growth rate 2.84 cm/year). **b** A 51 year-old-man, on August 2009 normal kidneys were found and on April 2013, 4.5 cm clear cell tumor was found (growth rate 1.23 cm/year). **c** A 45 year-old man on February 2006 normal kidneys were found and on November 2010, a 4 cm chromophobe carcinoma was found (growth rate 0.84 cm/year). **d** A 64-year-old lady that had left nephrectomy on May 2009. CT done on October 2010 showed normal right kidney. On August 2011 the patient had a 2.8 cm clear cell carcinoma (growth rate 3.36 cm/year)

The growth rate of renal cancer was calculated in 46 patients that had prior cross-sectional imaging study showing normal kidneys. Patients’ and tumors’ characteristics are presented in Table 1. Several examples of imaging studies showing normal kidneys followed by imaging of the same patients showing kidney cancer are presented in Fig. 1. After a median post-operative follow-up of 68 months, 5 patients (10.8 %) developed distant metastases. A total of 17 patients (36.9 %) died. In 3 patients (6.5 %) death was disease specific.

**Table 1:** Baseline characteristics of the patients

Characteristic	
Mean patient age [years, (SD)]	64 (11)
Gender	
Male	29 (%)
Female	17 (%)
Mean time from normal imaging to diagnosis [months (SD)]	33.6 (17.9)
Mean tumor’ largest diameter [cm, (SD)]	4.6 (2.5)
Histological type	
Clear cell	29 (63 %)
Papillary	14 (30.4 %)
Chromophobe	3 (6.5 %)
Fuhrman’s grade^a^	
1–2	14 (52 %)
3–4	13 (48 %)

^a^Patients with Fuhrman’s grade 2–3 were not included in the analysis

**Fig. 2:**
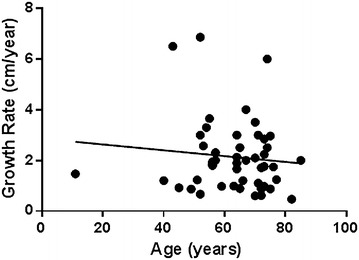
Correlation between patient’s age and tumor growth rate

Based on the model, the average growth rate of kidney tumors in the study was 2.13 cm/year (SD 1.45 cm/year, range 0.2–6.5 cm/year). The effect of various parameters on tumor growth rate is presented in Table 2. Figure 2 demonstrates that tumors’ growth rate correlates inversely with patient’s age (p = 0.007). Patient’s gender, type of previous imaging showing normal kidney, Fuhrman’s grade and metastases development during follow-up did not show significant correlation with growth rate.

**Table 2:** Growth tumor rate according to patient’s and tumor’s characteristic

Characteristic	Number of patients	Average growth rate (SD) cm/year	P value
All patients	46	2.13 (1.45 cm)	–
According tumor diameter	46	–	0.002
According to age	46	–	0.007
According to gender			0.97
Males	17	2.14 (1.4)	
Females	29	2.15 (1.5)	
According to type of imaging			0.197
Ultrasonography	28	2.35 (1.7)	
CT	18	1.85 (0.9)	
According to Fuhrman’s grade^a^			0.8
1–2	14	2.4 (1.05)	
3–4	13	2.25 (1.65)	
According to recurrence of tumor			0.5
Yes	5	2.68 (2.15)	
No	41	2.05 (1.3)	

^a^Patients with Fuhrman’s grade 2–3 were not included in the analysis

## Discussion

Understanding the natural history of solid tumors requires knowledge of their growth kinetics. Most of the data on the growth kinetics of kidney tumors is driven from surveillance studies of small enhancing masses. The leading studies are presented in Table 3. The average growth rate in most studies has been observed at 0.3 cm/year. When large tumors were followed, the growth rate was slightly higher (0.44–0.57 cm/year) (Mues et al. 2010; Mehrazin et al. 2014). In the current study, of younger, fit patients with pathologically confirmed cancer, the average growth rate was much larger at 2.13 cm/year.

**Table 3:** Leading studies in surveillance of renal cell carcinoma and observational studies

Author	Study type	Number of patients	Length of follow-up (months)^a^	Mean tumor diameter^a^ (cm)	Growth rate (cm/year)
Chawla et al. (2006)	Surveillance study	234	34	2.6	0.28
Jewett et al. (2011)	Surveillance study	127	29	2.1	0.13
Lee et al. (2008)	Surveillance study	30	12.3	2.6	0.59
Abouassaly et al. (2008)	Surveillance study	110	24	2.5	0.26
Crispen et al. (2008)	Surveillance study	109	26	2	0.21
Mason et al. (2011)	Surveillance study	82	36	2.3	0.25
Patel et al. (2012)	Surveillance study	71	34	2.2	0.21
Li et al. (2012)	Surveillance study	32	46	2.14	0.8
Brunocilla et al. (2014)	Surveillance study	58	88.5	2.6	0.7
Mues et al. (2010)	Surveillance study	36	36	7.13	0.57
Mehrazin et al. (2014)	Surveillance study	65	38.9	4.9	0.44
Staehler et al. (2010)	Observational study	9	14.6	2	6.4
Current study	Observational study	46	33.6	4.6	2.13

^a^In some of the studies the median and not the mean are reported

How is it possible to explain this multiplicity? We believe that differences in population and in study design can explain the diversity. While patients in surveillance studies are often at their 8th and 9th decades (Chawla et al. 2006; Mues et al. 2010), mean patient’s age in the current study was 64 years. As seen in Fig. 2, renal cancer growth rate correlates inversely with patient’s age. Therefore, a growth rate of a few millimeters per year may not apply to young patients.

Another explanation for the differences in growth rates between surveillance studies and the current study can be found in the Gompertzian model of tumor growth (Laird 1965). The model assumes an early exponential tumor growth followed by increasing retardation of the rate as the tumor matures and depletes its resources. This is reflected in a sigmoidal growth curve. While tumors in the current study were by definition in the early phase of their growth (proved by the normal imaging in the beginning of the period), tumors in the surveillance studies were diagnosed in various phases of their growth, some if not most of them beyond the inflection of the growth curve, where tumor growth rate is very slow. Additionally, a slow growth rate of tumors in surveillance studies is predicted by the length bias (Black and Welch 1993). Since most of the tumors reported in surveillance trials were diagnosed incidentally with cross-sectional imaging, they are inherently slow growing tumors. Indeed, 89 % of the tumors in the meta-analysis by Chawla et al. had histologic evaluation which demonstrated low grade, small tumors. (Cambell et al. 2007). In contrast, the “clinically significant” tumors in the current study were much larger and half had a high Fuhrman grade.

Suggestions for much faster growth rates of “clinically significant” renal tumors can be identified in the published literature. Staehler et al. reported on 9 patients that had kidney imaging 6 months or more prior to the diagnosis of renal cancer showing no tumor or a small tumor that was overlooked. They calculated an extremely high growth rate of 6.4 cm per year (Staehler et al. 2010). Oda et al. studied the growth rate of kidney cancer metastases and showed that it can reach 7.87 cm/year (Oda et al. 2001).

Despite its merits, the current study has is not devoid of limitations. It is retrospective, small and single institutional series. The two assumptions used for calculating tumor’s growth rate are not based on literature. Assuming that macroscopic tumor growth commenced shortly after the normal imaging however, is very conservative. If one was to hypothesize that the growth of the tumor began in the middle of the time period between the normal imaging and the diagnosis of the tumor would significantly increase the growth rate. Moreover, assuming that the growth is linear is certainly wrong, but considering the sigmoidal shape of the Gompertzian curve, the middle part of the curve is almost liner. Another concern is the combining of patients with initial normal ultrasonography and initial normal CT. Both modalities however, are acceptable for diagnosis and follow-up of kidney cancer and patients that had initial CT or initial ultrasonography had comparable cancer growth rates (Table 2).

## Conclusions

The growth rate of “clinically significant” renal cancer appears to be greater than the rate reported from active surveillance studies of enhancing renal masses. Further investigation is warranted for renal lesion growth dynamics, particularly for younger patients and those in neoadjuvant or screening trials.
